# Barriers and facilitators to implementing a longitudinal dementia education programme into undergraduate healthcare curricula: a qualitative study

**DOI:** 10.1186/s12909-021-02632-9

**Published:** 2021-04-09

**Authors:** Yvonne Feeney, Stephanie Daley, Breda Flaherty, Sube Banerjee

**Affiliations:** 1 Department of Neuroscience, Brighton and Sussex Medical School, Centre for Dementia Studies, Trafford Centre for Medical Research, University of Sussex, Falmer, Brighton, BN1 9RY UK; 2 Department of Medical Education, Brighton and Sussex Medical School, Watson Building, University of Brighton, Falmer, Brighton, BN1 9PH UK; 3 Faculty of Health, University of Plymouth, Plymouth, Devon, PL4 8AA UK

**Keywords:** Dementia education, Curricular change, Barriers, Facilitators, Leadership, Healthcare education, Longitudinal

## Abstract

**Background:**

As the numbers of people with dementia worldwide rises, there is a need for improved knowledge and awareness about the condition across the healthcare workforce. There are concerns that traditional models of healthcare education, which focus on short-term episodes of care, limit student understanding of long-term conditions. We therefore designed and delivered the Time for Dementia programme at five Universities in the UK. Through longitudinal contact with families living with dementia, healthcare students gain increased understanding about the experiences of living with dementia. However, implementing new educational models brings challenges. To enable implementation of similar programmes in other educational institutions, this study aimed to identify the common barriers and facilitators of implementing these types of longitudinal programmes at scale.

**Methods:**

To understand the facilitators and barriers of implementing a longitudinal dementia educational programme, a qualitative study was completed. Between October and December 2018, twelve in-depth semi-structured interviews were completed with university teaching staff (*n* = 6), programme administrators (*n* = 4), and Alzheimer’s Society staff (*n* = 2) that had key responsibilities for implementing Time for Dementia. Interview questions explored participants experiences, the facilitators, and the challenges encountered when implementing the programme. Interviews were audio recorded, transcribed verbatim, and analysed using inductive thematic analysis.

**Results:**

The analysis identified five key themes: “Leadership characteristics”, “Organisational and student buy-in”, “Perceived value and motivating factors”, “Team coalition and support”, and “Time and fit”. Implementation of the programme was enhanced by resilient leaders managing the challenges of curricular change. Their belief in the value of the programme, stakeholder buy-in, and supportive team working enabled challenges to be overcome. Workload was reduced and student buy-in increased as time progressed and as more resources became available. A flexible approach to implementation was recommended to ensure the programme fits within the established curriculum.

**Conclusion:**

Curricular change is a challenging task, yet necessary, if we are to improve care for people with long term conditions such as dementia. This study highlights the common barriers and facilitators experienced when implementing a longitudinal educational programme at scale. The findings presented in this study can be used by other educational institutions to manage curricular change efforts.

## Background

Currently, over 885,000 people have dementia in the UK [1] with an estimated 46.8 million people worldwide [2]. Dementia is a progressive disorder with a range of different aetiologies that can affect all areas of cognitive functioning, and which cause progressive functional limitation. The inherent presence of multi-morbidity and advancing age means that people with dementia are high users of health and social care services and have an increased risk of hospital admission [3, 4]. With at least one quarter of hospital beds occupied by people with dementia [5], and the high consultation rates in community and social care, most healthcare professionals, regardless of speciality, will encounter people with dementia during their career. This means that there is a need for a healthcare workforce with an understanding of the condition, how it affects individuals, and how it impacts on the treatment of other illnesses. Unfortunately, acute hospital care for people with dementia is currently associated with delayed or poorly planned discharge, and higher risk of falls, and delirium [6–8]. Poor outcomes and lack of awareness about the condition have resulted in policy directives [9–13] highlighting the need for a dementia-informed healthcare workforce, with the need for improvements in undergraduate training [14, 15].

Educational initiatives provided at undergraduate level could enhance student knowledge and attitudes towards dementia, however, there is a lack of evidence and policy direction about the type of dementia education required [16–18]. Traditionally, undergraduate education has been delivered through a series of acute placements and lecture-based learning. There are concerns that the transient nature of these encounters limit student understanding and the development of core values needed to care for people with dementia and other long-term conditions [19–21]. Taking an alternative approach, longitudinal educational models have demonstrated positive learning outcomes and good levels of student satisfaction [22, 23]. Based on person-centred care approaches, these models allow continuity in student learning through longitudinal contact with patients [24, 25]. Condition-specific educational models are less common, but evaluations of small-scale elective programmes have reported enhanced medical student understanding about dementia through longitudinal contact with people in the community; with improvements in knowledge, empathy, and positive attitudes towards dementia [26, 27].

Working in partnership with Alzheimer’s Society and local NHS trusts, the Time for Dementia (TfD) programme [28] was designed to increase healthcare students understanding of and attitudes toward dementia through longitudinal contact with a person with dementia and their family in the community. It was introduced in 2014 at Brighton and Sussex Medical School (BSMS) and the University of Surrey for medical, paramedic, and nursing students. Positive data on the impact of the scheme on student approaches to, knowledge of, and attitudes towards dementia [29, 30] have led to the programme’s expansion to other Universities (University of Brighton, Canterbury Christchurch University, and University of Greenwich), and its extension to other disciplines (physiotherapy, occupational therapy, radiography, and speech and language therapy students). The Time for Dementia programme is a mandatory component of the curriculum at participating universities. Between 2015 and 2020, over 4181 students have taken part in the programme.

Whatever the emerging evidence of the value of such approaches, implementing new educational models into undergraduate healthcare programmes requires change to an established curriculum, and such change is complex [31]. There are uniquely challenging elements associated with fitting a longitudinal programme into existing systems with a need to deliver across academic years. We have been unable to identify any research which has empirically investigated the practical facilitators and barriers of implementing undergraduate condition-specific longitudinal programmes. However, studies have reported barriers and facilitators when implementing more general Longitudinal Integrated Clerkships (LICs) [32–35]. A recent review of the literature [36] reported a range of recommendations to consider when implementing LICs. These include the need for effective leadership, proactive management of change efforts, collaborative working practices, maintaining a clear vision, and the need for adequate resources. Engaging with faculty staff and students when implementing longitudinal programmes was strongly recommended. An understanding of the motivational factors that drive individuals to engage in change efforts is also likely to be of value. It is suggested that individuals who are intrinsically motivated (e.g. interested in improving medical education) are more successful in implementing change than those motivated by extrinsic factors alone (e.g. pressured to implement a new programme) [37].

These findings can help understand the factors involved in longitudinal curricular change. However, there are significant differences between LICs and programmes like TfD. Both include a longitudinal component, however, LICs are traditionally designed for medical students that actively participate in patient care, and are under the supervision of a clinician [35]. It is therefore likely that condition-specific models like TfD may have unique barriers and facilitators in addition to those found in other longitudinal initiatives.

To support the future implementation and spread of such programmes, the aim of this study was to identify the barriers and facilitators encountered when implementing a longitudinal dementia educational programme as part of the core curriculum.

## Methods

### Study design

A qualitative study was designed and carried out in 2018 across the five universities that had implemented TfD. This study was underpinned by an interpretivist paradigm. Interpretivism subscribes to constructivism [38] and the researchers ontological and epistemological stance comes from the understanding that reality is subjective, uniquely individual, and socially constructed. Peoples’ experiences shape our understanding of a problem, and through narrative means, greater insight can be gained from their perception of their own reality. Therefore, this study used individual in-depth interviews to gather and understand the subjective experience of implementing TfD [39].

### Sample

Participants were invited to take part if they had experience in the implementation or oversight of TfD in one of the five universities. Eligible participants included three groups: university lecturers, TfD administrators, and TfD family network staff. University lecturers were tasked with implementing the programme at a faculty or discipline specific level and held primary teaching roles in medicine, nursing, physiotherapy, and occupational therapy. TfD administrators based within the universities managed the co-ordination of student visits. TfD network staff included a manager and support workers that co-ordinated the recruitment of families and supported universities during implementation and oversight of the programme. Thirteen participants were identified as eligible and were initially approached via email. Of those, twelve individuals responded to the email expressing their interest. These individuals were followed up and all agreed to take part in an in-person interview.

### Procedure

Semi-structured in-depth interviews were carried out between October and December 2018. Face to face interviews were arranged at a time and place to suit participants. Participation was voluntary and informed written consent was obtained. Socio-demographic data was collected, and interviews completed. A topic guide was developed following a review of the literature exploring curricular change and the implementation of longitudinal healthcare programmes. To assess its suitability, the topic guide was reviewed by two individuals with experience in leadership, change management, and implementing change. The topic guide explored the role of participants in their organisation and with the programme, the factors that enhanced implementation, challenges to internal delivery, and challenges to wider delivery. Interviews were digitally recorded using an encrypted recorder and lasted between 30 and 75 min. Interviews were transcribed verbatim and checked for accuracy by the lead author. Following this, audio files were deleted. A non-identifiable code was assigned to each transcript, and identifiable data was removed. All study data was stored on password protected university computers. Field notes were completed following visits documenting the setting and reflections.

### Data analysis

Data analysis was supported using QSR NVivo Version 11 software [40] to collate data. An inductive approach using thematic analysis was completed using Braun and Clarke’s [41] six steps. The first two transcripts were independently coded by two researchers, which involved giving descriptive labels to meaningful segments of text. Both researchers met to identify areas of overlap and disagreement in coding, until agreement was achieved. The remaining ten transcripts were analysed by the lead author. Codes were brought together to identify overarching themes, and thematic saturation was achieved when no new codes were identified in the data [42]. Meetings took place with two researchers to discuss, refine, separate, and discard themes, and to identify relationships across the data set [41]. During these meetings, the lead author was able to reflect on her own position within the research. To ensure rigour and reflexivity, field notes were completed, and team meetings held to discuss reflections. The Consolidated Criteria for Reporting Qualitative Research was used in the reporting of the study [43].

### Ethical approval

This study was approved by the Health Research Authority, National Research Ethics Service, NRES Committee London-Queens Square, reference number: 15/LO/0046.

## Results

### Sample

Twelve participants took part in the study. Their mean age was 45.8 years (SD 9.8), 83% of the sample were university based, and all participants were female. Participant demographics are described in Tables 1 and 2 provides details of the type of participant, their discipline, and responsibilities.

**Table 1:** Study participant demographic and occupational characteristics

Variables	Mean	(SD)	(Range)	n	%
**Age**	45.8	(9.8)	(28–61)	12	
**Sex**					
Female				12	100
**Ethnicity**					
White British				12	100
**Time spent working in current job role (months)**	54.9	(66.5)	(8–216)	12	
**Job role on TfD**					
TfD Manager				1	8.3
TfD Support worker				1	8.3
University Faculty Lead				3	25.0
University Lecturer				3	25.0
TfD Administrator				4	33.3
**Time spent working on TfD (months)**	24.0	(17.5)	(3–60)	12	
**Employing Organisation**					
Alzheimer’s Society				2	17.0
University				10	83.0

**Table 2:** Participant type and responsibilities on Time for Dementia

Type of Participant	Primary employment location	Primary job role	Main responsibilities implementing and overseeing the Time for Dementia programme (TfD)
**TfD Manager (*n*** **= 1)**	Alzheimer’s Society	TfD programme Family network staff	Manage recruitment of families for the programme, provide guidance and support to university leads and lecturers, manage safeguarding issues, plan student introduction and preparation sessions, correspond with families, attend site meetings.
**TfD Support worker (*n*** **= 1)**	Alzheimer’s Society	TfD programme Family network staff	Family recruitment, support to deliver student introduction and preparation sessions, manage family and student concerns, correspond with families, act as main link for a named university to provide updates and advice.
**University faculty lead (*n*** **= 3)**	University setting	Primary lecturing roles: Nursing: (*n* = 2) Medicine: (*n* = 1) Secondary role: implementing and oversight of TfD	Alongside primary teaching role: manage implementation of TfD, manage budgets, recruit administrator, work alongside university lecturer to plan implementation, update higher management, manage risk assessment.
**University lecturer (*n*** **= 3)**	University setting	Primary lecturing roles: Nursing: (*n* = 1) Occupational therapy: (*n* = 1) Physiotherapy: (*n* = 1) Secondary role: implementing and oversight of TfD	Alongside primary teaching role: integrate programme into curriculum, manage student timetable and plan visits, liaise with placement teams and wider faculty, manage student compliance issues, work with Alzheimer’s Society staff to plan and facilitate student sessions, support students with concerns, continued oversight of programme once implemented.
**TfD administrator (*n*** **= 4)**	University setting	University based administrator for TfD: (*n* = 4)	Co-ordinate student allocation to families, correspond with students about visits and updates, correspond with families at the start and end of TfD, monitor student compliance, attend, and minute site meetings, maintain student visit logs and family visit logs, work closely with Alzheimer’s Society staff to plan and problem solve.

### Qualitative themes

Five overarching themes were identified: “Leadership characteristics”, “Organisational and student buy-in”, “Perceived value and motivating factors”, “Team coalition and support”, and “Time and fit”. Table 3 outlines these themes and the facilitating factors and barriers identified for each.

**Table 3:** Barriers and facilitators implementing Time for Dementia

Theme	Facilitating factors for implementation	Barriers towards implementation
**Leadership characteristics**	Traits: commitment, ability to lead, confidence, persuasive, ability to build trust, clarity of role and responsibility, Acceptance of challenges, leadership within TfD team, decreasing workload over time.	Apprehension due to unfamiliar tasks, lack of protected time in combination with primary job role, additional workload alongside primary job role.
**Organisational and student buy-in**	External networking, shared vision, organisational buy-in, wider faculty and higher management buy-in, time increases student buy-in, peer to peer influence, practical methods to increase engagement.	Student engagement, student buy-in, wider faculty buy-in.
**Perceived value and motivating factors**	Invested interest, positive attitudes, pride, intrinsic value influenced by personal/ professional values, extrinsic values influenced motivation, valuable opportunity, limitations of traditional learning, value to family, value to the organisation, community involvement, network opportunities, external recognition.	Student motivation.
**Team coalition and support**	Supportive environment, TfD team support, team commitment and enthusiasm, collaborative teamwork, TfD team experience, ongoing support following implementation, effective communication, shared learning.	Blurring of defined roles, clarity of responsibilities, multi-organisational working, logistics.
**Time and fit**	Workload stabilised over time, student engagement increases with time, confidence grows, iterative learning, TfD team experience grows with implementation, dedicated administrator employed and in position, resources available, flexible implementation approaches, appropriate fit within curriculum.	Conflicting priorities, lack of protected time, limited resources during early stages of implementation, timing hotspots, crowded curriculum.

### Theme 1: leadership characteristics

Confidence was viewed as an important leadership trait facilitating implementation and day to day running of TfD. The ability to take ownership of the programme, instil confidence at an organisational level, manage challenging issues, and have appropriate authority to make curricular decisions was deemed as centrally important. Key university staff were identified as being responsible for implementing TfD at an organisational level and embedding the programme into their respective curriculum. These staff reported clear understanding of their responsibilities, however some felt apprehensive during the implementation phase due to uncertainty about the task ahead. These feelings were intensified by a lack of time and additional workload associated with the change effort. However, resilient attitudes and acceptance of these challenges facilitated positive approaches and problem-solving.

*“I think the other thing is you’ve got to be really proactive; you’ve got to be willing to realise there are going to be challenges and problems, but actually you’re going to work round them and you’re going to make it happen…” (Participant 12)*

### Theme 2: organisational and student buy-in

The importance of organisational buy-in was highlighted as both a facilitating factor and a barrier to implementing TfD. The importance of building a vision and gaining support from the senior faculty members, for example the Vice Chancellor, helped to increase organisational ownership and recognition of the programme’s importance. Raising awareness of the programme amongst wider faculty was identified as a challenge given the limited time available. However, this was seen as a necessity as the wider faculty could have a pivotal role in influencing student buy-in.

*“I think it’s that whole buy-in about people (other lecturers) championing the project, about championing it for students as well, because ultimately these lecturers will be personal tutors to these students. So, if a personal student comes and says “Oh, I’m doing this dementia project and I don’t like it,” if the lecturer doesn’t understand what it’s all about, they might agree with the student, they might send the wrong messages...” (Participant 11)*

Engaging students was challenging in the initial phases of implementation. However, proactive approaches enhanced student engagement. This was achieved through tailored introduction and preparation sessions prior to the programme beginning. The involvement of families living with dementia and student advocates attending these sessions helped increase student buy-in.

*“I think them seeing the families engaging with them, kind of when they start their studies, I think it just helps to take the mystery out of it.” (Participant 4)*

As more students completed the programme, student buy-in was enhanced over time through peer-to-peer influence and acceptance that TfD was part of their curriculum.

*“ … with the first two or three cohorts, they weren’t always as engaged as they should have been … this year we had a special session with one of the families for them, that were just telling from their experience about having the student visits and also their daily lives with dementia. I think that actually, it clicks with the students … ”* (Participant 4)

### Theme 3: perceived value and motivating factors

Improving dementia education was identified as a high priority and all participants discussed their belief in the value of TfD as a way of achieving this. Their vested interest in this priority motivated their decision to be involved in the programme. This sense of value originated from personal and professional experiences and their understanding of the limitations of traditional dementia education.

*“I think personally, I was very excited, but I think there was a general excitement about being involved in something that was so innovative … actually, to incorporate the voice of people with dementia, you know, it really, for me, has made it more meaningful.”* (Participant 3)

Participants reported that their belief in the value of the programme was a driving force in motivating them to implement TfD despite the challenges. They reported a sense of positivity and pride that the programme was successfully implemented within their university. Some felt the innovative aspects of TfD were highly valuable to their organisation, demonstrating progressive and modern approaches to curricular delivery and being at the forefront for change. Recognition of the programme importance through awards and media interest was viewed by some to add value, as was the opportunity for networking opportunities with colleagues from other universities implementing the programme.

“ … *the fact of being involved in something with different universities, having those contact points with different universities and contact points with the Alzheimer's Society, which I probably wouldn’t have had, if it hadn’t have been for the project, so actually now having those... that networking side of it, I think is also really beneficial* … *” (Participant 12)*

### Theme 4: team coalition and support

Team coalition (through the stakeholders involved in the programme) was a critical element to the implementation of TfD. Team commitment and enthusiasm was felt to be a driving force behind the ability to manage perceived and actual challenges. A dedicated support TfD team consisting of BSMS and Alzheimer’s Society staff, provided guidance to each university in the implementation of TfD. Support was provided through regular on-site meetings, and the provision of templates and resources, for example student facing documents and teaching plans. This support was perceived by participants as essential, and paramount in alleviating anxiety about the implementation process. Knowledge and expertise within the supporting team were reassuring for participants.

*“I think that having a visit from [supporting TfD Team member] made some of those anxieties significantly reduced, because she was quite confident and, you know, she was very much like “we’ve done this before, you know, it’s fine”. So, I think that that helped.” (Participant 9)*

Close working relationships between the supporting team and individual universities enabled collaborative working across organisations. However, as confidence grew following implementation, the need for more clarity in roles was highlighted.

### Theme 5: time and fit

Unsurprisingly, the early stages of implementation were identified as being the most taxing for participants. A lack of protected time or dedicated resources, such as administrative support, increased workload for university lecturers. Some participants felt administrators need to be employed and in position earlier in the implementation phase.

*“ … having that* (administration*) support in early and having your resources there and making sure … all your ducks in a row before you even entertain the idea of introducing it to the students … .” (Participant 8)*

Once appointed, administrators helped to reduce workload and time pressures.

Implementing a new educational model into an already crowded curriculum was recognised as being difficult. Caution about the programme’s fit was identified to ensure it was positioned appropriately within the curriculum and made sense to students, for example, within an existing curriculum module with similar objectives. For one site, implementing the programme during a scheduled periodic review of the curriculum facilitated its implementation.

*“ … we just basically looked at these are the modules, and it fits well with and so it meant our course underwent a periodic review, so it’s a five-year review process every course goes under, so as part of that review process then we incorporated the TfD within those modules … ” (Participant 8)*

To facilitate implementation, a degree of flexibility was suggested for new sites.

*“ … implementing TfD in a new university has to have a degree of flexibility … because actually if it doesn’t fit within your curricula, it doesn’t fit, and I think there has to be that degree of flexibility with how it is actually implemented and introduced within universities.” (Participant 3)*

In general, participants agreed that once implemented the challenges reduced significantly. With the passing of time, student engagement increased, wider faculty awareness improved, and the programme was well recognised in the organisational context.

## Discussion

This study aimed to identify the barriers and facilitators encountered when implementing a longitudinal educational programme, at scale, into existing undergraduate healthcare programmes. Five key themes were identified: “Leadership characteristics”, “Organisational and student buy-in”, “Perceived value and motivating factors”, “Team coalition and support”, and “Time and fit”.

Leadership traits were evident throughout this study’s findings. This is unsurprising given the complexities of managing curricular change [31]. Facilitating the implementation of programmatic change such as TfD requires leadership [36] that can share a collaborative vision, and those that can motivate and inspire others [44, 45]. Leaders needed to instil confidence and trust at an organisational level and be aware of the importance of raising awareness of the programme throughout their School or Faculty. Leaders also needed to hold a clear understanding of their responsibilities, be committed to its implementation, and believe in the value of the programme. These factors appeared to facilitate a sense of resilience. Although many were apprehensive at the early stages, this resilience motivated them to deal with perceived and actual challenges. Resilient attitudes are important to enable leaders to learn, grow, and bounce back during challenging situations [46]. Participant vision [34, 36], coupled with a strong sense of urgency [33, 47] to improve dementia education, influenced their decision to adopt the programme. All of those interviewed in this study were motivated to implement the programme, however, this raises a question as to how to develop this approach in other universities so that leaders’ values and vision are congruent with the programme.

The degree of compatibility and complexity into its implementation is an important consideration for universities [48]. Participants identified the need for programme fit within the already crowded curriculum. A longitudinal programme such as TfD needs to be embedded across modules with comparable learning outcomes, such as those that address Long-Term Conditions [49]. All universities have different demands on their curriculum; one size will not fit all. Therefore, flexible approaches need to be taken to find the most suitable local fit [34]. Mirroring other longitudinal educational models [22, 33], early stages of implementation were identified as the most challenging due to lack of resources, lack of administrative staff, and additional workload alongside primary lecturing roles. However, our findings are consistent with literature from other longitudinal programmes [22, 50, 51], namely, that workload and time pressures reduced and stabilised as time progressed and resources were made available. New sites planning to implement a similar programme need to account for the burden on faculty leads and lecturers’ time. Providing protected time is a necessary facilitator to manage the additional responsibilities of implementing and overseeing such programmes alongside their primary teaching roles. Appointing a dedicated administrator before the programme is implemented reduces this burden.

Motivation is considered a persuasive factor toward the willingness to change [45]. The relationship between intrinsic and extrinsic motivation can positively or negatively impact individuals’ basic psychological needs towards self-determination [52]. A significant finding from this study is the demonstration of participants’ intrinsic motivation to implement TfD despite the challenges. This was influenced by personal and professional core values such as participants’ own experiences of dementia care in healthcare settings. While it is argued that intrinsic motivation is a crucial element in educational change efforts [37], perceived value may be insufficient to enhance motivation in isolation. This study found buy-in from the organisation and support from an experienced team enhanced motivational efforts despite the challenges faced. Considered selection of faculty leads and lecturers that are intrinsically motivated to implement the programme may facilitate change efforts. However, good support needs to be provided by the organisation to foster and maintain their intrinsic motivation. A range of extrinsic influences (external, introjected, identified, and integrated regulation factors [52]) can also impact motivational efforts during implementation. Faculty leads and lecturers that are influenced by extrinsic factors alone (e.g., pressure to implement the programme), may have less motivation to achieve the task especially if effective support is not provided. To facilitate implementation efforts, new sites should understand the factors that impact intrinsic and extrinsic motivation [37] and develop clear plans to support motivational efforts.

Successfully managing curricular change depends on effective team working [47], and the implementation of TfD was perceived to be dependent on the combined efforts of a coalition. Team coalition facilitated the implementation of TfD through the motivation of a shared goal; to ensure the programme was implemented and run effectively. An environment that fails to nurture external motivators through the lack of supportive conditions risks eroding motivation and can impact individuals’ feelings of autonomy, competence, and relatedness; ultimately stifling motivation [52, 53]. Support, reassurance, and guidance from the TfD team along with their expertise and knowledge were viewed as a facilitating driver toward supporting university leaders to address those psychological needs. It is not clear what impact the lack of such support might mean for a new university implementing the programme.

A collaborative environment and human resource development are necessary to manage curricular change. Internal network support was viewed as a necessary part of this, and participants identified senior management, wider faculty and student buy-in as important agents to establishing and maintaining a collaborative environment [31]. Resistance to change is not uncommon [36, 47], however, this was not found in this study. Participants spoke of factors that may help mitigate organisational resistance, however, they did not report resistance from the wider faculty or organisation. They did however encounter difficulties ensuring the wider faculty were made aware of TfD due to limited time. Mirroring other longitudinal models [32, 33], support from the senior faculty was instrumental in raising organisational awareness. Wider faculty awareness was deemed a facilitator as other staff members may need to reinforce student compliance with the programme at times, particularly because it will span different modules. Lack of time to raise awareness amongst the wider faculty [33] can limit buy-in, however, this study found the passing of time helped increase wider faculty engagement and awareness of the programme and increased recognition and acknowledgement that it formed a core part of the curriculum. Where external drivers are incongruent with intrinsic values, motivational efforts can be hampered or fail altogether [52]. This may explain why participants identified early challenges when engaging students.

Students had little autonomy of decision-making about taking part in TfD as it was a mandatory part of their undergraduate education. However, efforts to foster extrinsic motivational factors by providing a supportive environment (peer to peer reassurance and wider faculty awareness) and socially contextual activities (preparation and introduction sessions), appear to have increased motivation and ultimately, buy-in. To enhance readiness and buy-in, students are introduced to TfD several months before the programme starts. A session lasting 1 h is facilitated by Alzheimer Society staff, a university lecturer, a family living with dementia, a student with previous experience of the programme, and a TfD administrator. The introduction session provides students with an overview of dementia, an overview of TfD, and family and student experience of the programme. This session also introduces students to members of the TfD team and provides key contact details.

In the month before the start of TfD, students attend a preparation session lasting 2–3 h. The session is facilitated by Alzheimer’s Society staff, a university lecturer, and TfD staff. Topics covered in the preparation session include further information on TfD, the purpose of the programme and its relevance to student learning, preparing for visits, safety considerations, the role of Alzheimer’s Society in the programme, and an interactive session to address the students’ hopes and concerns. Finally, a handbook is provided to all students and contains information that includes advice about visiting families, suggested topics to explore, recommended structure for visits, what to do if issues arise, and key contact details.

The student body must be considered as a powerful coalition member and it is imperative that universities build student buy-in as a facilitating factor when implementing the programme.

### Strengths and limitations

There are two main limitations to this study. First, all participants were female and of similar ethnic origin, although this was indicative of the overall population of the teams involved in the implementation of TfD at the time of data collection. This may have limited the generation of themes and further research is needed to include a diverse sample as the programme is implemented in new sites.

Second, this study found resistance to change was not commonly experienced by participants from the wider faculty or organisation. This suggests their organisation may already have had a culture more conducive to and accepting of change. It may be that this represents the possibility that the universities involved were ‘innovators’ and/or ‘early adopters’ and so might differ from the later categories (early majority, late majority, and laggards) of adopters at institutions less primed for this change [54]. Therefore, some institutions may have greater resistance to change.

However, it is a strength that TfD was implemented by universities delivering health professional undergraduate training in Kent, Surrey, and Sussex, so there was no selection based on willingness to participate within the region, aiding generalisability. When presented with the possibility of participation, with the costs covered by Health Education England, all the universities in the region cooperated enthusiastically. A further strength is that the good quality qualitative approach used here has generated novel and practical guidance about the implementation of a condition specific longitudinal model in universities.

## Conclusion

Good quality dementia education is a priority for the healthcare workforce. Recognising the limitations of traditional undergraduate dementia education, five universities in the UK have taken a progressive approach by implementing a novel dementia longitudinal educational programme. Managing curricular change can be daunting and a challenge, however with the right preparation and leadership in place, the task is readily achievable. Committed leaders that believed in the value of the programme facilitated its implementation, and efforts were enhanced by organisational buy-in and team coalition building. Programme fit, managing time, and availability of resources during the implementation phase requires careful consideration in new implementation sites. Findings from this study should be useful for universities planning similar longitudinal programmes.

## Data Availability

The datasets (interview transcripts) used and analysed during the current study are not publicly available due to concerns regarding compromise of participant confidentiality.

## Abbreviations

BSMS: Brighton and Sussex Medical School
TfD: The Time for Dementia Programme
